# Species Diversity, Structure, and Regeneration Status of Woody Plants in Saleda Yohans Church Forest, South Wollo, Ethiopia

**DOI:** 10.1155/2023/3853463

**Published:** 2023-04-17

**Authors:** Amare Bitew Mekonnen, Ali Seid Mohammed, Aster Demissew

**Affiliations:** Bahir Dar University, Department of Biology, Bahir Dar, Ethiopia

## Abstract

The study was conducted to determine woody plant species composition, structure, and regeneration status in Saleda Yohans Church forest, South Wollo Zone, Ethiopia. Five transect lines oriented to north-south directions at a distance of about 500 m were laid down along transects across the forest. Fifty main plots of 20 m × 20 m were laid for tree/shrub data collection. Within each main plot, 5 small quadrats of 5 m × 5 m were laid in the corners and centre for the collection of seedling and sapling data of woody species. All vegetation data within the plots were counted and recorded. Tree height and diameters at the breast height of plants were also measured and estimated. Moreover, frequency, basal area, diversity, evenness, and other vegetation data were analyzed. The result showed that this Church forest is composed of 50 woody plant species distributed in 31 families. The Shannon–Wiener diversity index and evenness values of the forest were found to be 3.82 and 0.84, respectively. *Lamiaceae* was the dominant family followed by *Fabaceae* in their species composition. The density of trees/shrubs, saplings, and seedlings was 625, 650, and 935 ha^−1^, respectively. Based on the result, the entire vegetation of Saleda Yohans Church forest is in good regeneration status. In conclusion, although this Church forest appears to be in a good regeneration status, its species richness is low compared to a similar study of some other vegetation. Therefore, attention should be given to the rehabilitation of this forest.

## 1. Introduction

The tropical forest is the most biologically diversified ecosystem with large species richness, the highest net primary production (NPP), and accumulating high above and below-ground biomass ha^−1^due to consistent availability of water, sunlight, and high temperatures in the region [1]. However, it is highly threatened by the net loss of forest due to high anthropogenic pressure [2]. Deforestation is still alarmingly high in the tropical region resulting in the formation of small patches of fragmented forests [3].

The forest cover in Africa is estimated to be 650 million ha, constituting 17% of the world's forest. Over 50% of African land is covered by Afromontane vegetation [4]. Ethiopia is a mountainous country with great geographic diversity. The diversified topographic features in Ethiopia made the country to have a rich biodiversity forest cover in tropical Africa [5]. This made Ethiopia to have the fifth largest floral diversity in tropical Africa [6]. This floral diversity is rich in endemic elements. About 6,027 vascular plant species (including subspecies) with about 10% endemism have been documented [7]. The forest resources of Ethiopia reported in [2] put Ethiopia among countries with a forest cover of 10–30%. According to this report, Ethiopia's forest cover (FAO definition) is 12.2 million ha (11%).

Churches have native vegetation rich in biodiversity hosting different plants and animals [8]. Church forests did not significantly decrease in size, rather these forests have remained remarkably resilient on the landscape despite decades of dramatic change to the world around them [9]. However, sacred groves have been disturbed and are vulnerable due to land use changes. Their indigenous trees and shrubs are reducing and being substituted by exotic tree plantations like *Eucalyptus* species because of people's preference for construction, firewood, cash source, and other economic uses [8, 9].

There are number of factors for deforestation and the decline or loss of biodiversity, and the problem is evident in Northern Ethiopia where forests are downscaled to few protected areas, especially in the compounds of Orthodox Tewahido Churches [10]. To this end, very little of the natural forest and wild animals remain today. These all are the results of a long-term human occupation of the area, accompanied by sedentary agriculture, extensive cattle husbandry, exploitation of biodiversity, extracting fuel, and the use of wood for construction [10, 11]. To overcome problems in biodiversity loss, the ministry of agriculture in collaboration with different national and international organizations is working to implement agro-forestry and community tree planting programs for the last three decades. Yet, the challenges of minimizing the rate of deforestation, due to the lack of appropriate technologies to improve conservation practices and the imbalance between the forest resource and the demand of the ever-increasing population of the country, remain unsolved [12, 13].

The only areas where one can observe forests or trees in Northern Ethiopia are in some protected areas, monasteries, and the Church surroundings, and hence these patches of biodiversity in the monastery and Church compounds are believed to survive as *in situ* conservation sites [12].

The study area is vulnerable to land degradation, due to continuously increasing anthropogenic activities, disturbances in the form of agricultural encroachment, firewood, and grazing in the extremely fragile and sensitive ecosystems of the Church forests. These factors have been critically threatening the natural resources to the extent of losing the unique combination of biodiversity. Sufficient scientific information is lacking on the vegetation of this Church forest. Therefore, the main objective of the study is to assess woody plant species diversity, structure, and regeneration status of forests and to give clear information for urgent conservation measures and sustainable use of the forest.

## 2. Materials and Methods

### 2.1. Description of the Study Area

**Figure 1:**
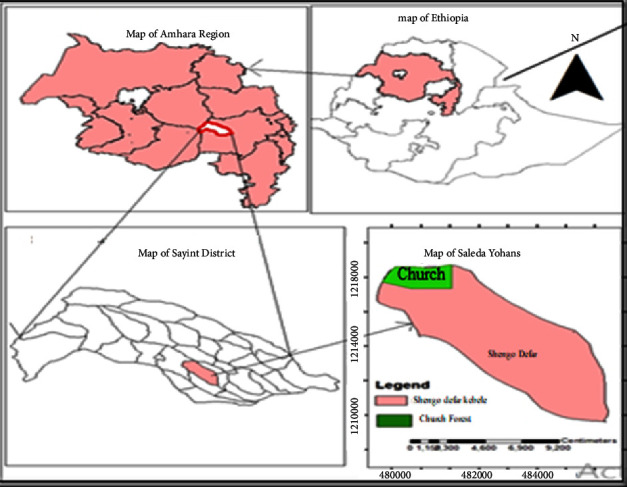
The study area map.

The study was conducted in Saleda Yohans Church forest found in Amhara Sayint District, South Wollo Zone, Amhara National Regional State, Western Ethiopia (Figure 1). The district is 590 km far from Addis Ababa and 189 km from Dessie. The highest point is Tabor Mountain with 3955 meter above sea level (masl), and Adijbar is the town of Amhara Sayint District [14]. The study area is geographically located at 11°01′29.77″N and 38°49′41.64″E.

The altitude of this district ranges from 500 masl at the bottom of the canyon of the Abay River to 3,955 masl which is the highest point of South Wollo Zone called Mount Tabor. The district consists of one small town kebele and 31 rural kebeles. Its physical landscape is mostly characterized by chains of mountains. The terrain feature of Sayint includes hot lowlands along the river side of Abay and Beshilo. The genesis of the soils in the study area is dominantly cambisols (61.61%), lithosols (cover 25.61%), and rocky 12.78% (the area is found mainly in mountainous regions, where the soil has been eroded to the extent that hard rock comes near to the surface) [14].

**Figure 2:**
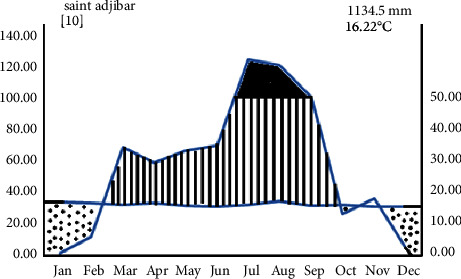
Climadiagram of the study area (2009–2018) (data source: [15]).

The rainfall and temperature condition of the study area was described based on the 10 years of data collected from 2010–2019 taken from the nearest town, Kombolcha Metrological Service Center. Accordingly, the study area obtains high rainfall between July and August and low rainfall from October to December. The dry season is between January and February, whereas the lowest monthly rainfall from the total is 9.31 mm recorded in December. The highest mean annual rainfall of the district is 1134.5 mm. The average annual maximum temperature of the study area is 16.22°C. The area also has a mean monthly minimum and maximum temperature of 7.6°C and 24.84°C, respectively (Figure 2).

### 2.2. Reconnaissance Survey

A reconnaissance survey was made in the study area in order to have general setting of the environment to estimate the position, number, and length of transects and plots to be laid across the forest. Schulz et al. [16] suggested that it is important to know the size of the vegetation as well as the number of plots to be laid out per hectare before data collection. Unless the spatial boundaries of the study site are separated and properly recognized, it is difficult to get accurate measurements and perform effective work.

### 2.3. Study Design

**Figure 3:**
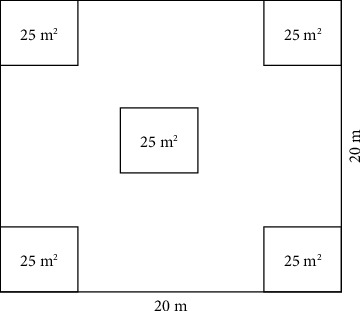
Layout of the main plot and subplot design.

Five transect lines oriented to north-south direction separated by a distance of 500 m were laid down. Then, 50 sample plots with size 20 × 20 m were sampled using a systematic random sampling technique along the five transect lines at 50 m intervals on each transect line [17]. The number of plots was determined by species accumulation curve where a point no longer adds extra new species encountered for given additional plot sampling efforts during the reconnaissance survey. In each main plot, 5 subplots with size 5 × 5 m were established one at each corner of the main plot and one at the center of the main plot to collect data related to seedlings and saplings (Figure 3). Woody plant species were classified into three size classes (seedlings, saplings, and mature plants) based on their height and diameter at breast height. In each sample plot, all adult woody individuals with height >2.5 m and DBH >2.5 cm were considered as adult plants; woody individuals with height between 1 m and 2.5 m were counted as saplings, and individuals with height <1 m were counted as seedling.

### 2.4. Methods of Data Collection

All trees/shrubs species found in the main plots and saplings and seedlings of woody species found in subplots were recorded with their densities, and voucher specimens were also collected with their vernacular names. The pressed and dried plant specimens were later identified using the authenticated and preserved specimen in Adds Ababa University National Herbarium by referring to [18] and flora of Ethiopia and Eritrea [19, 20]. For those woody species with DBH >2.5 cm, circumference at breast height (1.3 m above the ground) was measured for later conversion into DBH. For those tree boles branched at 1.3 m, measurements were taken separately and averaged. The height of the individual wood plants was also measured using a meter-marked stick.

### 2.5. Data Analysis

Further diversity data including frequency, species richness, Shannon–Wiener diversity index (H′) and equitability/evenness index (J), dominance, population structure, and regeneration status were extracted and analyzed as follows.

#### 2.5.1. Diversity Data Analysis

The Shannon–Wiener diversity index (H′) was calculated using the following equation [17].

(1) $$\begin{array}{c} H'=-\overset{s}{\underset{i=1}{\sum }}Pi\left(In\left(Pi\right)\right)\text{,} \end{array}$$

where *H*′ = Shannon diversity index, *s* = the number of species, Pi = the proportion of individuals of the *i*^th^ species expressed as a proportion of total cover in the sample, and ln = the natural logarithm.

Species richness was calculated by summing up the number of woody plant species identified in the sample plot. Shannon's evenness or equitability index was calculated as follows [17]:

(2) $$\begin{array}{c} J=\frac{H^{\prime }}{Hmax}=\frac{H^{\prime }}{\ln\left(S\right)}\text{,} \end{array}$$

where *J* = evenness; *H*′ = Shannon–Wiener diversity index; *H*max = ln(S); and *S* = total number of species in the sample. The value of evenness index falls between 0 and 1.

Density of woody species was calculated by converting the total number of individuals of each woody species encountered in all the quadrats across all transects used in the site to equivalent number per hectare [17].

(3) $$\begin{array}{c} Density=\frac{number\;of\;above\;ground\;stem\;of\;species\;counted}{sample\;area}\text{.} \end{array}$$

Frequency of each woody plant species was computed as the proportion (%) of plots (quadrats) within which a species is found out of the total number of quadrats used in the site.

(4) $$\begin{array}{c} Frequency=\frac{number\;of\;plots\;in\;which\;that\;species\;occur}{total\;number\;of\;plots}\text{.} \end{array}$$

#### 2.5.2. Structural Data Analysis

Diameter at breast height (DBH) was obtained from the measured circumference as follows:

(5) $$\begin{array}{c} DBH=\frac{CBH}{\pi }=\frac{C}{\pi }\text{.} \end{array}$$

DBH was calculated from circumference of each adult woody species using formula D = *C*/*π*, where DBH = diameter at breast height, *C* = circumference, and *π* = constant with value 3.14.

Dominance of the woody species was determined from basal area obtained by converting DBH data to basal area as follows. That is, BA = *π* d^2^/4, where BA = basal area in m^2^ per hectare, *d* = diameter at breast height, and *π* = 3.14159265.

(6) $$\begin{array}{c} Therefore,dominance=\frac{total\;basal\;area}{area\;of\;sample\;in\;ha}\text{.} \end{array}$$

(i) The importance value index (IVI) was obtained by summing up relative frequency, relative density, and relative dominance, i.e., IVI = relative density + relative frequency + relative dominance [17].
  (7) $$\begin{array}{c} \text{Where},\;Relative\;density=\frac{density\;of\;species\;A}{total\;density\;of\;all\;species}\times 100\text{,}\\ relative\;frequency=\frac{frequency\;of\;species\;A}{total\;frequency\;of\;all\;species}\times 100\text{,}\\ relative\;dominance=\frac{dominance\;of\;species\;A}{total\;dominance\;of\;all\;species}\times 100\text{.} \end{array}$$

#### 2.5.3. Regeneration Status Data Analysis

The population structure and regeneration status were derived based on height class vs. density of each height class and by comparing individual number of seedlings with saplings and saplings with mature trees/shrubs [21]. Regeneration status was assigned as follows: “good,” if the number of seedlings > saplings > mature strata; “fair,” if the number of seedlings > saplings < mature strata; “poor,” if the number of mature > saplings > seedling strata; “none,” if a species is absent, both in sapling and seedling stages, but present as mature; and “new”, if a species has no mature, but only sapling and/or seedling stages.

## 3. Results and Discussion

### 3.1. Woody Species Composition

A total of 50 woody plant species distributed in 31 families were identified in Saleda Yohans Church forest (Table 1). Of 50 species, 14 or 28% were shrubs, 4 (8%) were shrubs/trees (mean found in both tree and shrub habit), and 32 (64%) were trees. The result showed that there is high proportion of trees than shurbs, which indicates the local people respect the sacredness of the forest and protect trees not to be cut for private use. *Lamiaceae* (5 species, 10%) was the most dominant family in the study area followed by *Fabaceae* (with 4 species, 8%) and *Euphorbiaceae* (with 3 species, 6%). The remaining 10 families were represented by 2 species (4%) each, and 18 families were represented by single species (2%) each (Table 2). Compared to some previous studies conducted in a similar ecosystem of Ethiopia, for example, Denkoro forest in South Wollo with 64 species [22], Menagesha Amba Mariam forest with 70 species in Central Shewa [9], and Gedo forest with 130 species in West Shewa [23], species richness of this study area is relatively low. However, species richness of this study area is greater than that of Yemrehana Krestos Church forest (39 species) in North Wollo [4] and Kurib forest (39 species) in Awi zone of Amhara National Regional State [24]. This difference may be due to differences in some environmental variables and anthropogenic effects.

### 3.2. Species Diversity and Evenness

The overall Shannon–Wiener diversity and evenness of the forest were 3.8 and 0.84, respectively. The value of Shannon–Wiener diversity index usually was found to fall between 1.5 and 3.5 and rarely surpassed 4.5 [25]. The Shannon diversity index values observed in Saleda Yohans Church forest falls in rare range. The result showed that Saleda Yohans Church forest is of good species diversity with the different species having uniform abundance. Wassie [26] described that high species diversity and evenness could be attributed to the presence of optimum environmental factors such as altitude, slope, and adequate nutrients and moisture. The high diversity of woody plants in Saleda Yohans Church forest was probably a result of high species richness and abundance of this study area. Comparison of the diversity value of Saleda Yohans Church forest with other studies conducted on other similar vegetation types of the country, Menagesha Suba Forest with 112 species (1992). (H′ = 2.57) [27] and Angada forest with 87 species (H′ = 3.4) [28], showed that Saleda Yohans Church forest is more diverse though found to be lesser in species number. The high diversity of woody plants in Saleda Yohans Church forest could be due to the less dominance of few species over the other, which could be attributed to low habitat disturbance and environmental conditions favoring all species more or less equally [29, 30].

### 3.3. Population Structure

**Figure 4:**
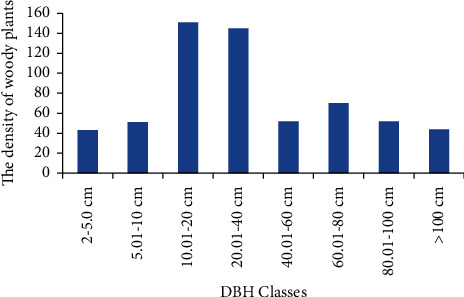
General DBH distribution patterns (structure) of Saleda Yohans Church forest.

The DBH distribution of woody plants was a bell-shaped pattern. This indicates a decrease when the class interval increased. But the densities of medium-sized species with DBH class 40−60 cm were greater than very few sized and large-sized species. Among the eight DBH classes, the medium sized DBH class species had the higher density than the smaller and higher DBH classes (Figure 4).

**Figure 5:**
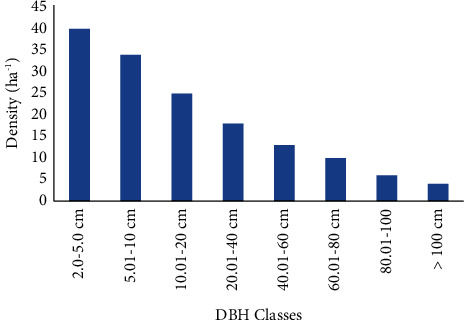
DBH distribution patterns (structure) of *Juniperus procera* in the Church forest.

The general DBH distribution pattern of some selected dominant woody species (*Juniperus procera*, *Olea europaea* subsp. *cuspidata*, and some others) of Saleda Yohans Church forest is inverted J-shaped distribution pattern (Figure 5). The DBH distribution pattern of some other selected dominant woody species (*Dodonaea angustifolia* and some others) is bell-shaped pattern. This indicates the density of medium-sized species was greater than small DBH sized and large DBH sized species.

This result is contrasted with the study of [23, 27] where the density of plant species decreased as the DBH classes increased and the density of species in lower DBH classes encountered more species than medium and higher DBH classes. They also stated that the density of species in lower DBH classes was decreased due to the browsing effect, environmental factors, the impact of large plants for resources, and agricultural expansion. The density of large plants is also affected mainly by anthropogenic activities such as fencing, fuel wood, and illegal wood harvesting for construction.

### 3.4. Density

From 50 quadrats, a total of 4420 individuals (1250 mature, 1870 seedlings, and 1300 saplings) of woody species were counted. The densest species of this study area was *Juniperus procera* species and followed by others such as *Acokanthera schimperi, Calpurnia aurea,* and *Allophylus abyssinicus* (Table 1). Compared to many other previous similar research studies in similar ecosystem, for example, that of Dodola forest [31], Wof Washa forest [32], Gemechis forest [33], Abebaye forest [34], Zengena forest [30], and Yegof forest [35], vegetation of the current study was less dense. Topographic, climatic, and anthropogenic variations occurring in different sites affect density of given vegetation.

### 3.5. Frequency

Frequency reflects the pattern of distribution and gives an approximate indication of the heterogeneity of a stand. The most frequent species in the study area were *Juniperus procera* (4.039%), *Olea europaea* subsp. *cuspidata* (3.32%), *Acokanthera schimperi* (2.96%), *Euphorbia abyssinica* (3.23%), *Carissa spinarum* (2.60%), *Allophylus abyssinicum* (2.42%), *Prunus africana* (2.42%), and *Acacia abyssinica* (1.61%). The least frequent species in the study area were *Otostegia integrifolia* (0.26*%*), *Ficus sur*as (0.62%), *Ficus vasta* (*0.8*%), and *Grewia ferruginea* (0.7%) (Table 1) [36]. As pointed out, species with the highest relative frequency have a wide range of seed dispersal mechanisms by wind, livestock, animals, and birds.

### 3.6. Basal Area

The overall basal area of woody plant species of the forest was 45.5 m^2^·ha^−1^. Among the woody plant species, *Juniperus procera* with a relative BA (14.54%) ranked first followed by *Olea europaea* subsp. *cuspidata* (14.35%)*, Acokanthera schimperi* (8.44%), and *Cupressus lusitanica* (8.36%). Plant species that have large basal areas are said to be most ecologically important (dominant) species in a given ecosystem.

*Allophylus abyssinicus, Prunus africana, Buddleja polystachya,* and others have less contribution for total basal area (Table 1). Species with the highest basal area do not necessarily have the highest density [37]. Basal area provides the measure of the relative importance of the species than simple stem count. If we consider density rather than basal area, shrubs could have been the dominant species. Species with the largest contribution in dominance value through the higher basal area could be considered the most important species in the study area.

### 3.7. Important Value Index

The value of IVI helps to make comparisons of species in their ecological significance. In this study, the highest IVI values were recorded for *Juniperus procera, Olea europaea* subsp. *cuspidata*, *Acokanthera schimperi, Cupressus lusitanica, Euphorbia abyssinica,* and *Carissa spinarum*. Species with a small contribution to the total IVI were *Aloe* sp.*, Ficus sur*, and *Ficus vasta* (Table 1). IVI value indicates the ecological importance of species in the community. The result is in line with the study of [4, 37] which reported that these species were considered as the leading dominant species in their study area. Woody species which have lower rank to IVI (*Jasminum abyssinicum, Pterolobium stellatum, Aloe* sp.*, Ficus vasta, Grewia ferruginea,* and *Ficus sur*) are more threatened and need immediate conservation.

### 3.8. Regeneration Status

A total density of 935 seedling individuals' ha-1, 650 sapling ha-1, and 625 mature individuals ha-1 were counted from all sample plots. Species such as *Juniperus procera, Acokanthera schimperi, Prunus africana*, and *Allophylus abyssinicus* have the largest contribution to the seedling counts per hectare. Species with largest contribution to sapling were *Calpurnia aurea, Myrica salicifolia, Olea europaea* subsp. *cuspidata,* and *Lippia adoensis*. Generally, in this seedling and sapling assessment, the number of seedling was greater than sapling and good regeneration was observed for most shrub species than trees. This could be due to the belief of local people to respect the sacredness of the church and protect it from animal grazing and cutting down trees in Church yard. The presence of good regeneration potential shows stability of the species to the environment, and the density values of seedlings and saplings are considered regeneration potential of the species [38].

**Figure 6:**
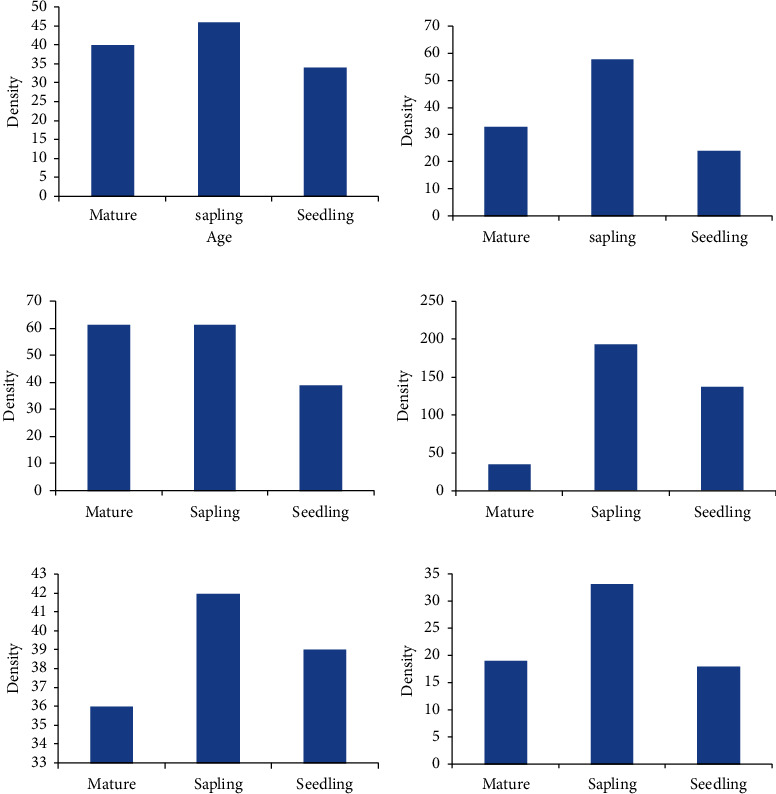
The regeneration status of some dominant plant species in the study area. (a) *Olea europaea*. (b) *Juniperus procera*. (c) *Myrica salicifolia*. (d) *Allophylus abyssinicus*. (e) *Carissa spinarum*. (f) *Acacia abyssinica*.

In the study area, some dominant plants species such as *Olea europaea, Juniperus procera, Acokanthera schimperi, Euphorbia abyssinica*, *Carissa spinarum,* and *Allophylus abyssinicus* were described according to their age structure (Figure 6). In all plant groups, the density of seedlings was greater than saplings and mature ages. *Olea europaea* plants have almost equal number of matures saplings and seedlings and they have higher seed dispersal with good regeneration status. From the selected dominant woody plants, both *Allophylus abyssinicus* and *Carissa spinarum* L. have more saplings than mature and seedlings and they are good indicator for regeneration status in the study area.

According to the report of Shambel [28], the presence of sufficient population of seedlings, saplings, and adults indicates successful regeneration of forest species and the presence of saplings of adult trees also indicates the future composition of a community. Regeneration status of trees can be predicted by the age structure of their population. Regeneration of a particular species is poor if seedlings and saplings are much less than the mature trees.

## 4. Conclusion and Recommendation

### 4.1. Conclusion

The study showed that a total of 50 species of woody plants were identified in the study forest. Among these, *Juniperus procera* was the most dominant tree in the study forest followed by *Olea europaea*, *Euphorbia abyssinica*, and *Acacia abyssinica* in the study area. On the other hand, *Rumex nervosus, Hypericum revolutum, Vernonia amygdalina,* and *Grewia ferruginea* are the least dominant woody plant species in Saleda Yohans Church Forest.

From the structural analysis, the density of tree individuals of Saleda Yohans Church forest and the densities of lower DBH class were decreased may be due to disturbances such as browsing, agricultural expansion, and competition with large plants for resources. The total basal area of woody plant species in Saleda Yohans Church forest is 45.5 m^2^ per hectare. *Juniperus procera* has the highest basal area followed by *Olea europaea, Prunus africana,* and *Euphorbia abyssinica* which have the highest basal area when compared to the other woody plant species in the study forest; therefore, it can be concluded that these trees are the most important tree species in the forest. From frequency analysis, *Juniperus procera* is the most frequent species followed by *Olea europaea* subsp*. cuspidata.* On the other hand, some of the species were with the least frequency. There is high percentage of number of species in lower frequency classes and low percentage of number of species in higher frequency class. This indicated the presence of high degree of floristic heterogeneity in the study forest.

### 4.2. Recommendations

Based on the results of the study, the following recommendations are forwarded.

Species with the lowest IVI like *Jasminum abyssinicum, Pterolobium stellatum, Aloe* sp.*, Ficus vasta, Grewia ferruginea,* and *Ficus sur* are the most threatened and need immediate conservation using *in situ* conservation strategies. Tree-growing project should be encouraged by the local communities with more emphasis of varieties of local woody plant species and the threatened species that need immediate conservation. All head priests, deacons, and followers together with the agricultural development office should work on sustainable utilization of the resources. Priests and followers of the Church should respect the Church's thoughts on the conservation of vegetation and raise their awareness on the values of forest resources and the ecological consequences of deforestation of forest around the study area. It is recommended to reduce anthropogenic disturbances to reduce the pressure put on the forest. We would also like to recommend further studies to be done on soil physicochemical properties, soil seed bank, and the status of herbaceous species in this Church forest.

## Figures and Tables

**Table 1:** List of woody plant species in Saleda Yohanes Church forest with their vegetation data.

No	Biological name	Family name	Habit	RD (%)	RF (%)	RDO (%)	IVI (%)
1	*Acacia abyssinica* Hochst Benth	*Fabaceae*	Tree	2.49	1.62	2.72	6.83
2	*Acokanthera schimperi* (A. DC.) Schweinf	*Apocynaceae*	Tree	3.39	2.96	8.44	14.79
3	*Albizia gummifra* (J.F.Gmel.) C.A.	*Fabaceae*	Tree	0.90	1.71	1.49	4.10
4	*Allophylus abyssinicus* (Hochst.) Radlk	*Sapindaceae*	Tree	3.05	2.42	2.82	8.29
5	*Aloe* sp.	*Aloaceae*	Shrub	1.11	1.67	0.01	2.79
6	*Argemone Mexicana* L.	*Papaveraceae*	Tree	1.95	1.53	0.24	3.72
7	*Becium grandiflorum* (Lam.) Pic. Serm.	*Lamiaceae*	Shrub	1.70	1.62	1.54	4.86
8	*Bersama abyssinica* Fresen.	*Francoaceae*	Tree	1.36	1.80	1.10	4.26
9	*Buddleja polystachya* Fresen.	*Loganiaceae*	Tree	2.15	1.80	2.64	6.59
10	*Calpurnia aurea* (Ait.) Benth	*Fabaceae*	Tree/shrub	2.04	2.69	1.92	6.65
11	*Carissa spinarum* L.	*Apocynaceae*	Tree/shrub	3.39	2.60	4.54	10.53
12	*Clutia lanceolata* Forssk.	*Euphorbiaceae*	Shrub	1.58	1.44	1.35	4.37
13	*Cordia africana* Lam.	*Boraginaceae*	Tree	1.13	1.89	2.61	5.63
14	*Croton macrostachyus* Del.	*Euphorbiaceae*	Tree	2.04	2.51	1.89	6.44
15	*Cupressus lusitanica* Mill.	*Cupressaceae*	Tree	3.51	2.60	8.36	14.47
16	*Discopodium penninervium* Hochst.	*Solanaceae*	Tree	1.58	1.89	0.17	3.64
17	*Dodonaea angustifolia* L.f.	*Sapindaceae*	Tree	1.76	1.44	0.07	3.27
18	*Dombeya torrida* (J.F.Gmel.) Bamps	*Sterculiaceae*	Tree/shrub	0.70	1.26	1.54	3.50
19	*Dovyalis abyssinica* (A. Rich.) Warb.	*Salicaceae*	Tree	2.26	2.15	2.72	7.13
20	*Echinops* sp. A. Rich.	*Asteraceae*	Tree	1.70	1.90	0.13	3.73
21	*Ekebergia capensis* Sparrm.	*Lamiaceae*	Shrub	1.92	1.62	2.61	6.15
22	*Eucalyptus globulus* Labill.	*Myrtaceae*	Tree	1.29	2.62	0.01	3.92
23	*Euclea schimperi* (A.DC.) Dandy	*Ebenaceae*	Tree	1.83	1.17	0.11	3.11
24	*Euphorbia abyssinica* J.F.Gmel.	*Euphorbiaceae*	Tree	3.62	3.23	6.01	12.86
25	*Ficus sycomorus* L.	*Moraceae*	Tree	0.68	1.76	0.04	2.48
26	*Ficus vasta* Forssk.	*Moraceae*	Tree	0.79	1.72	0.06	2.57
27	*Grewia ferruginea* A.Rich.	*Tiliaceae*	Tree	1.31	1.22	0.03	2.56
28	*Heteromorpha arborescens* (Spreng.) Sch.	*Apiaceae*	Tree	1.72	1.97	0.11	3.80
29	*Hypericum revolutum* Vahl	*Hypericaceae*	Tree	1.58	1.67	0.03	3.28
30	*Jasminum abyssinicum* L.	*Oleaceae*	Shrub	1.11	1.55	0.33	2.99
31	*Juniperus procera* L.	*Cupressaceae*	Shrub	6.79	4.04	14.53	25.36
32	*Justicia schimperiana* Hochst.	*Acanthaceae*	Tree	2.94	2.24	2.72	7.90
33	*Laggera tomentosa* Schultz Bip.	*Compositae*	Shrub	1.72	1.62	1.49	4.83
34	*Lippia adoensis* Hochst. ex Walp.	*Verbenaceae*	Shrub	1.47	2.24	0.01	3.72
35	*Maytenus arbutifolia* (A. Rich.) Wilczek	*Celastraceae*	Tree/shrub	2.38	2.24	2.72	7.34
36	*Myrica salicifolia* A. Rich.	*Myricaceae*	Tree	1.47	1.71	0.33	3.51
37	*Ocimum lamiifolium* Hochst. ex Benth.	*Lamiaceae*	Shrub	1.70	1.97	0.31	3.98
38	*Olea europaea* L. subsp. *cuspidata* Cif.	*Oleaceae*	Tree	4.52	3.32	14.35	22.19
39	*Otostegia integrifolia* Benth.	*Lamiaceae*	Shrub	1.56	1.68	0.04	3.28
40	*Phytolacca dodecandra* L. Herit.	*Phytolaccaceae*	Shrub	1.13	2.62	0.18	3.93
41	*Prunus africana* (Hook.f.) Kalkman	*Rosaceae*	Tree	2.94	2.42	2.78	8.14
42	*Pterolobium stellatum* (Forssk) Brenan	*Fabaceae*	Tree	1.45	1.35	0.13	2.93
43	*Rosa abyssinica* R.Br.	*Rosaceae*	Tree	2.22	2.42	0.28	4.92
44	*Rotheca myricoides* Var.	*Lamiaceae*	Shrub	1.74	1.69	0.04	3.47
45	*Rumex nervosus* Vahl.	*Polygonaceae*	Shrub	1.47	2.57	0.17	4.21
46	*Salix subserrata* Willd.	*Salicaceae*	Tree	1.58	1.97	0.27	3.82
47	*Solanecio gigas* (Vatke) C. Jeffrey	*Compositae*	Tree	1.81	2.69	0.18	4.68
48	*Solanum marginatum* L.f.	*Solanaceae*	Shrub	1.13	1.97	1.92	5.02
49	*Urera hypselondendron* A. Rich.	*Urticaceae*	Tree	1.13	1.89	0.03	3.05
50	*Vernonia amygdalina* Del.	*Asteraceae*	Tree	0.93	1.62	1.89	4.44

RD, relative density; RF, relative frequency; RDO, relative dominance; IVI, important value index.

**Table 2:** List of woody plant families with their number of genera and species in Saleda Yohans church forest.

No	Family	Genera	No. of species	Percent
1	*Acanthaceae*	1	1	2
2	*Aloaceae*	1	1	2
3	*Apiaceae*	1	1	2
4	*Apocynaceae*	2	2	4
5	*Asteraceae*	2	2	4
6	*Boraginaceae*	1	1	2
7	*Celastraceae*	1	1	2
8	*Compositae*	2	2	4
9	*Cupressaceae*	2	2	4
10	*Ebenaceae*	1	1	2
11	*Euphorbiaceae*	3	3	6
12	*Fabaceae*	4	4	8
13	*Francoaceae*	1	1	2
14	*Hypericaceae*	1	1	2
15	*Lamiaceae*	5	5	10
16	*Loganiaceae*	1	1	2
17	*Moraceae*	2	2	4
18	*Myricaceae*	1	1	2
19	*Myrtaceae*	1	1	2
20	*Oleaceae*	2	2	4
21	*Papaveraceae*	1	1	2
22	*Phytolaccaceae*	1	1	2
23	*Polygonaceae*	1	1	2
24	*Rosaceae*	2	2	4
25	*Salicaceae*	2	2	4
26	*Sapindaceae*	2	2	4
27	*Solanaceae*	2	2	4
28	*Sterculiaceae*	1	1	2
29	*Tiliaceae*	1	1	2
30	*Urticaceae*	1	1	2
31	*Verbenaceae*	1	1	2
	Total	50	50	100

## Data Availability

The data used for analysis of this study are included within the article.
